# Poor adherence to antiretroviral therapy among adult people living with HIV initiated during the COVID-19 epidemic waves – observations at the University Teaching Hospital in Lusaka, Zambia

**DOI:** 10.3389/fpubh.2023.1094214

**Published:** 2023-03-13

**Authors:** Powell Kafwanka, Flavia Muyinza Nalule, Charles Michelo

**Affiliations:** ^1^Department of Epidemiology and Biostatistics, School of Public Health, University of Zambia, Lusaka, Zambia; ^2^Department of Internal Medicine, School of Medicine, University of Zambia, Lusaka, Zambia; ^3^Strategic Centre for Health Systems Metrics (SCHEME), Lusaka, Zambia; ^4^Global Health Institute, Nkwazi Research University, Lusaka, Zambia

**Keywords:** detectable viral load, COVID-19, adult PLWHIV, ART adherence, U=U, UTH

## Abstract

**Background:**

Coronavirus disease 2019 (COVID-19)-related disruptions in healthcare services and clinical outcomes have been predicted and documented. However, little is known about how antiretroviral therapy (ART) adherence disruptions caused by the COVID-19 pandemic have manifested amidst the ‘Undetectable = Untransmittable' campaign initiative. Using a patient's viral load as a proxy for medication adherence, our study aimed to determine the adherence to ART on first-line medications among adult people living with human immunodeficiency virus (PLWHIV) at the University Teaching Hospital in Lusaka, Zambia during the pandemic.

**Methods:**

This was a hospital-based cross-sectional study. Secondary data of PLWHIV registered to receive ART from the Adult Infectious Disease Centre was extracted from the SmartCare^®^ electronic health record system to constitute a resultant data set that this study used. The data extraction form was used to extract values of dependent (ART adherence measured by viral load detectability) and independent variables and imported them into the statistical analysis tool, STATA version 16.1 MP. Descriptive statistics of individual characteristics, testing for associations using Pearson's chi-square test, and stratified and combined multivariable logistic regression were performed.

**Results:**

Of the 7,281 adult PLWHIV included in this study, 9.0% (95% CI 8.3–9.6%) were virally detectable. Estimates of the odds ratios of detectable viral load remained significantly higher among adult PLWHIV who were initiated on ART after the U=U campaign was launched in Zambia and were on a monthly 2.51 (1.31–9.03) or 6-monthly 4.75 (3.52–6.41) dispensing of a dolutegravir-based regimen and those on 6-monthly dispensing of an efavirenz-based regimen 4.67 (2.16–10.08) compared to their counterparts. Overall estimates showed us the same picture 4.14 (3.22–5.31), having adjusted for all other predictor variables.

**Conclusion:**

We found that a high proportion of people with detectable viral load in the study population, irrespective of medication refill interval and type of regimen, was concentrated among adult PLWHIV who started treatment during the COVID-19 epidemic waves, as compared to those who started treatment before the pandemic. This observed disparity suggests the inherent impact of the pandemic on the adherence to ART among adult PLWHIV in Lusaka, Zambia. This further illustrates how exposed program responses are to external shocks, especially in already weakened health systems, and the need to create program response buffers and resilient program-specific strategies to minimize the effect of external disruptions.

## 1. Introduction

Globally, 37.7 million people were living with HIV (PLWHIV) in 2020, out of which 36 million were adults. According to the statistics, 1.5 million were new infections, and 680,000 people died from AIDS-related illnesses in 2020 (1). HIV/AIDS remains a major public health issue worldwide, having caused the death of almost 32 million people, and infected over 74 million people, with the largest numbers recorded in low and middle-income countries (2). UNAIDS estimated that, in 2020, over 54% (20.6 million) of the total PLWHIV in the world were from sub-Saharan Africa (SSA), which accounted for 800,000 people with new infections and 310,000 people who died from AIDS-related illnesses that year. Only 11.7 million people were accessing antiretroviral therapy (ART) (3) .

Despite global targets to eliminate AIDS as a public health threat by 2030, ~14% (3.5 million) of the 25.4 million PLWHIV on ART are virally non-suppressed with the majority in SSA (1). Therefore, in 2016, the Prevention Access Campaign, a health equity initiative to end the HIV/AIDS pandemic as well as HIV-related stigma through the use of treatment as prevention (TasP), launched the ‘Undetectable equals Untransmittable' (U=U) initiative (4). The scientific evidence for U=U came from studies conducted at the time (HPTN 052, PARTNER, Opposites Attract, and PARTNER 2) that demonstrated that PLWHIV on ART for at least 6 months and who take medication daily as prescribed and achieve and maintain an undetectable viral load, defined as a viral load of fewer than 200 copies of HIV RNA per milliliter of plasma, have effectively no risk of sexually transmitting the virus through unprotected vaginal or anal sex to their HIV-uninfected sexual partners (5).

TasP through the U=U initiative has proven to be effective in attaining the UNAIDS 95-95-95 target. The target aimed to diagnose 95% of all HIV-positive individuals, provide ART for 95% of those diagnosed, and achieve viral suppression for 95% of those on treatment by 2025 (6). Attaining this will subsequently lead to ending the HIV pandemic by 2030 through effective reduction of the risk of HIV transmission and improving the quality of life for the PLWHIV by reducing the stigma associated with the disease and by motivating efforts to become virally suppressed (7). A study conducted in 25 countries, including South Africa, further found that being aware of the U=U message through discussions with healthcare workers was associated with higher odds of undetectable viral load in PLWHIV (8).

The Zambia Demographic and Health Survey (2018) reported that 1.2 million people in Zambia were PLWHIV, which corresponded to 11.1% of adults aged 15–49 years (9). In Zambia, U=U message campaigns have been incorporated into numerous public health campaigns and clinical guidelines since their launch in May 2019 to achieve HIV/AIDS epidemic control by 2030 (10). Despite Zambia adopting the U=U strategy for the prevention of HIV transmission in mid-2019, the burden of HIV prevalence and transmission remains high as evidenced by 46,000 new infections among adults and HIV-associated mortality estimated at 17,200 for the year 2020 (9). Moreover, HIV infection prevalence was estimated to be highest in Lusaka province, where ~340,000 persons were found to be infected (11).

Amid these national programmatic efforts for HIV response, COVID-19 hit the world in 2019. COVID-19, an infectious disease caused by severe acute respiratory syndrome coronavirus 2 (SARS-CoV-2), was first identified in Wuhan, China in December 2019 and has since spread rapidly around the world (12). The outbreak was characterized as a Public Health Emergency of International Concern (PHEIC) on 30 January 2020 and later as a pandemic on 11 March 2020 by the WHO soon after Zambia implemented the U=U campaign initiative as a strategy to use treatment as prevention (TasP) to achieve HIV/AIDS epidemic control by 2030 (13). However, the implementation of quarantine, social distancing, and community containment measures during the COVID-19 pandemic reduced access to routine HIV services, such as access to ART medications, as these measures restricted transportation and limited people's ability to travel to obtain their ART refills (14). PLWHIV require daily doses of ART medications to achieve and maintain an undetectable viral load and subsequently reduce the risk of forward transmission (7). Therefore, disruptions in routine HIV services can hinder adult PLWHIV's ability to adhere to ART medications, leading to an increased risk of having a detectable viral load and subsequently increasing the risk of HIV transmission, especially in SSA where most adult PLWHIV are found coupled with limited access to resource-constrained health facilities.

Given the high burden of HIV prevalence and transmission in Zambia, especially among adult PLWHIV in Lusaka, we postulated that, despite having launched the U=U campaign initiative 3 years ago, the COVID-19 pandemic might have affected both the campaign and adherence to ART medications among adult PLWHIV at the University Teaching Hospital (UTH) in Lusaka, Zambia. Several recent studies have looked at the potential impacts of the COVID-19 pandemic on HIV-related outcomes. However, little is known about the extent to which the COVID-19-related disruptions in adherence to ART have materialized especially amid the U=U campaign initiative. Therefore, we aimed to determine the adherence to ART among adult PLWHIV on first-line ART medications at the UTH in Lusaka, Zambia during the COVID-19 pandemic.

## 2. Materials and methods

### 2.1. AIDC, UTH, Zambia

The center is located at the University Teaching Hospital, a tertiary hospital in Lusaka, Zambia, which serves as a referral center for both private and other public health facilities in the country. We used data from the Smartcare electronic health record (HER) system of AIDC. The majority of the more than 10,000 adult (≥ 18-year-old) patients served at the center were receiving HIV care and ART from the facility. The center, among other services, specializes in the provision of comprehensive HIV and AIDS prevention, care, and treatment services through outpatient and inpatient services. The main facility has a pharmacy, consultation rooms for clinical reviews, and a modern laboratory infrastructure with the ability to perform virology and other tests for infectious diseases. AIDC serves a large portion of the HIV-positive population in and around Lusaka, and thus the center's patients represent a variety of different demographic groups. The center is one of a growing number of facilities in sub-Saharan Africa with a well-established Smartcare EHR system. It houses both demographic and clinical information about all PLWHIV who receive HIV care services at the center. We restricted our analysis to those who had been on ART for at least 6 months and were currently on a first-line ART regimen.

### 2.2. COVID-19 and PLWHIV study

#### 2.2.1. Design

Data for this study stem from the Adult Infectious Disease Centre (AIDC) database. A hospital-based cross-sectional study was conducted among adult PLWHIV registered to receive ART from the University Teaching Hospital's Adult Infectious Disease Centre (AIDC). The dataset contained patients who accessed ART services from AIDC between June 2000 and December 2021. All documented records of adult (≥18 years) PLWHIV receiving care at the AIDC at UTH in Lusaka, Zambia who had been on ART for at least 6 months prior to 31 December 2021 with a documented most recent 2021 viral load result as of 31 December 2021 were included in the study. However, patients without available data on the ART start year, dispensation interval, CD4 count at ART initiation, and current type of ART regimen were excluded. A complete enumeration of all individuals receiving care at the UTH AIDC who were on ART for at least 6 months, and with a documented most recent 2021 viral load result prior to 31 December 2021 was done, giving a total of 7,281 study participants who met the abovementioned inclusion criteria. The World Health Organization defines adherence to medication as the degree to which the person's behavior corresponds with the agreed recommendations from a health care provider (15). Furthermore, adherence to ART has been found to be a principal determinant of viral suppression (16). It is for this reason that in this study adherence to ART was defined using a patient's viral load as a proxy for adherence. Therefore, a detectable viral load (viral load ≥200 copies/ml) signified poor adherence to ART. Residual HIV transmission risk persists during the first 6 months of ART, meaning that for U=U to hold, someone has to be on ART for at least 6 months in order to achieve an undetectable viral load (17).

#### 2.2.2. Data extraction

**Figure 1:**
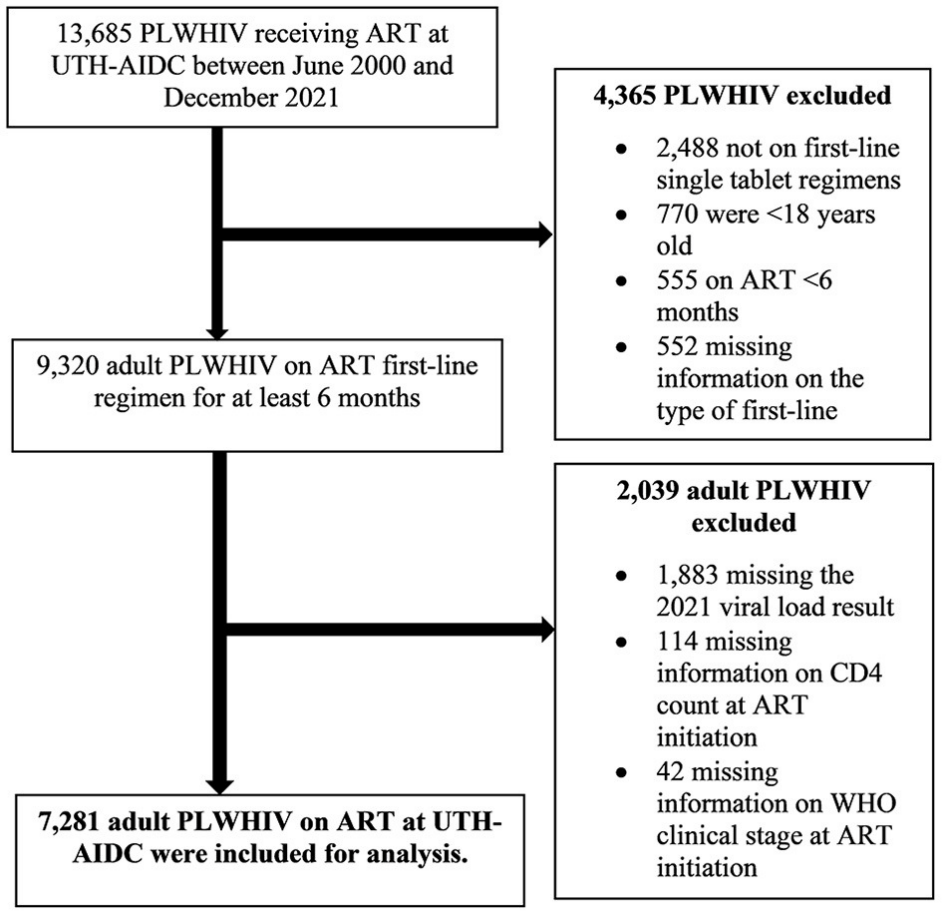
Study participants' flowchart of adult PLWHIV on ART at the AIDC, UTH, Lusaka.

Anonymized or de-identified individual patient data on sociodemographic variables, clinical data, treatment variables, and treatment outcomes (viral load result) of the 7,281 adult PLWHIV who had met the set inclusion criteria as shown in Figure 1 were extracted from the SmartCare^®^ electronic health record system in Microsoft^®^ Excel^®^ and imported into STATA^®^ version 16.1 MP (Stata Corporation, College Station, Texas, USA). Records, containing variables of interest, were generated in Microsoft^®^ Excel^®^ where they were formatted, cleaned, merged, coded, and imported into STATA^®^ version 16.1 MP (Stata Corporation, College Station, Texas, USA) in preparation for analyses.

#### 2.2.3. Statistical analysis

Data were extracted from the SmartCare^®^ electronic health record system in Microsoft^®^ Excel^®^ and imported into STATA^®^ version 16.1 MP (Stata Corporation, College Station, Texas, USA) for analyses. The normality of continuous variables was checked using graphs (histogram) and q-q plots, and the mean and standard deviation were reported if the normality assumption was satisfied. Otherwise, the median and interquartile range were reported. Some of the continuous variables were transformed into categorical variables to improve the interpretability of results and account for non-linear associations. Descriptive statistics using absolute frequencies with proportions for categorical variables were reported stratified by viral detectability. Comparisons between viral detectability and predictor variables among adult PLWHIV were achieved using Pearson's Chi-square tests for proportions (replaced by Fisher's exact test for sparse data).

Furthermore, logistic regression modeling stratified by the type of first-line ART regimen and ART dispensation interval was used to estimate and quantify the impact of the COVID-19 pandemic on the adherence to ART (measured by viral detectability) having adjusted for potential confounders using the following variables: age, sex, CD4 cell count at ART initiation, and WHO clinical stage at ART initiation. The statistical significance level was set at a *p*-value < 0.05 and a 95% confidence interval.

### 2.3. Ethical considerations

AIDC is mandated to collect routine data on all infectious diseases, including HIV/AIDS and COVID-19. The data are available for continued analysis to inform programming. However, for our study ethical clearance was sought and obtained from the University of Zambia Biomedical Research Ethics Committee (UNZABREC) (REF. No. 2099-2021), and the National Health Research Authority (NHRA) (NHRA0000005/20/11/2021). Permission to extract de-identified data from the SmartCare^®^ EHR was obtained from the Ministry of Health Headquarters, the Lusaka Provincial Health Office (LPHO), the Lusaka District Health Office (LDHO), and from the management of the UTH-Adult Hospital and the AIDC. The study did not require participants to be directly involved because the information was extracted from SmartCare^®^ EHR. As such, patients were not required to give their time and other resources to participate in this study because only their already documented records were used. Participants' confidentiality was maintained throughout by the exclusion during data extraction of participants' unique identifiers such as name, surname, patient ART folder number, and identity number.

## 3. Results

### 3.1. Demographic and clinical characteristics

Table 1 shows the demographic and clinical characteristics of eligible adult PLWHIV included in our study stratified by viral load detectability. Overall (*n* = 7,281) mean age was 43.9 years (± 12.8) and the majority (60.8%) were women. Of the 7,281 adult PLWHIV, 9.0% (95% CI 8.3–9.6%) were virally detectable (viral load ≥200 copies/ml). In terms of gender comparison, females were more likely to have a detectable viral load than male patients, however, this association was statistically not significant (*p*-value = 0.93). Of the participants, 5.9% (430) were initiated on ART after the U=U campaign initiative was launched in Zambia as opposed to those who started ART before the launch of the campaign. Furthermore, viral detectability, a proxy for poor adherence to ART, was significantly higher among those who initiated ART during the pandemic [114/430 (26.5%) vs. 539/6851 (7.9%)], while 11.8% (861) presented with advanced HIV disease (AHD: CD4 cell count < 200 cells/mm^3^) at the initiation of ART. Moreover, most of the adult PLWHIV (78.5%) were on a dolutegravir-based single tablet as the first-line ART regimen, and the majority were on a 6-monthly dispensing of ART medications (84.0%) and were initiated on ART at WHO HIV clinical stage 1 (64.3%).

**Table 1:** Demographic and clinical characteristics of adult PLWHIV by viral detectability at AIDC of UTH, Lusaka, Zambia (*n* = 7,281).

**Characteristic**	**Detectable**	**Undetectable**	**Overall**
	**VL**≥**200 copies/ml**	**VL**<**200 copies/ml**	
	**% (** ***n*** **)**	**% (** ***n*** **)**	**(** ***n*** **)**
	**9.0% (653)**	**91.0% (6,628)**	***n** **=*** **7,281**
**Sex**			
Male	8.9% (255)	91.1% (2,602)	2,857
Female	9.0% (398)	90.0% (4,026)	4,424
Age in years Mean (SD)	40.7 (13.1)	44.2 (12.7)	43.9 (12.8)
**CD4 cell count at ART initiation**			
≥200 cells/mm3	7.4% (478)	92.6% (5,942)	6,420
< 200 cells/mm3	20.3% (175)	79.7% (686)	861
**ART start year**			
< 2019	7.9% (539)	92.1% (6,312)	6,851
2019+	26.5% (114)	73.5% (316)	430
**Current type of ART regimen**			
Dolutegravir-based (TLD/TafED)	7.7% (439)	92.3% (5,275)	5,714
Efavirenz-based (TLE/Atripla)	13.7% (214)	86.3% (1,353)	1,567
**Dispensing interval**			
1 month	17.6% (105)	82.4% (492)	597
3 months	11.0% (63)	89.0% (508)	571
6 months	7.9% (485)	92.1% (5,628)	6,113
**WHO staging at ART initiation**			
WHO HIV clinical stage 1	8.8% (414)	91.2% (4,270)	4,684
WHO HIV clinical stage 2	11.9% (65)	88.1% (479)	544
WHO HIV clinical stage 3	8.8% (117)	91.2% (1,207)	1,324
WHO HIV clinical stage 4	7.8% (57)	92.2% (672)	729

1. Overall mean age in years is 43.9 (±12.8), among those with detectable viral load, 40.7 (±13.1), and for those with undetectable viral load, 44.2 (±12.7). 2. Overall median CD4 cell count at ART initiation in cells/mm^3^ (IQR) is 461 (306-635), among those with detectable viral load, 343 (190-541), and for those with an undetectable viral load.

### 3.2. Viral detectability by U=U campaign categories

To determine the impact of the COVID-19 pandemic on adherence to ART, the predictor variable of interest was the patient's ART start year, categorized as either having initiated ART before the U=U campaign was launched in Zambia in 2019 (< 2019) or starting ART after its launch in Zambia (2019+), a period that was largely characterized by the COVID-19 pandemic. Table 2 compares the levels of viral load detectability between these two categories stratified by the type of first-line regimen they were currently on and the ART dispensing interval. The majority (67.0%) of the adult PLWHIV included in this study were on a 6-monthly dispensing of a dolutegravir-based first-line regimen with the least (1.8%) being those who were on a 3-monthly dispensing of efavirenz-based first-line regimen. A higher proportion of adult PLWHIV with a detectable viral load was consistent among those who started ART after the U=U campaign initiative was launched in Zambia, a period largely characterized by the COVID-19 pandemic. However, this difference in the proportions of viral detectability was only statistically significant among patients who were on either a monthly or 6-monthly dispensing of a dolutegravir-based first-line regimen, and a 6-monthly dispensing of efavirenz-based first-line regimen. When we combined both regimens, this association between the patient's ART start year and viral detectability was only statistically significant among patients who were on a monthly and 6-monthly dispensing of ART.

**Table 2:** Viral detectability (detectable vs. undetectable) by U=U campaign categories among adult PLWHIV receiving ART at the AIDC of UTH in Lusaka, Zambia, stratified by type of first-line ART regimen and dispensing interval.

**Type of regimen**	**ART start year**	**Dispensing interval (Months)**					
		**One**		**Three**		**Six**	
		**Detectable row% (** ***n*** **)**	**Undetectable row% (** ***n*** **)**	**Detectable row% (** ***n*** **)**	**Undetectable row% (** ***n*** **)**	**Detectable row% (** ***n*** **)**	**Undetectable row% (** ***n*** **)**
TLD/TafED *n =* 5,714	< 2019 2019+	*n =* 394		*n =* 439		*n =* 4,881	
		13.5 (50) 34.8 (8)	86.5 (321)^f*^ 65.2 (15)	7.3 (29) 15.9 (7)	92.7 (366)^f^ 84.1 (37)	5.8 (265) 24.9 (80)	94.2 (4,295)^c*^ 75.1 (241)
TLE/Atripla *n =* 1,567	< 2019 2019+	*n =* 203		*n =* 132		*n =* 1,232	
		22.3 (44) 50.0 (3)	77.7 (153)^f^ 50.0 (3)	19.4 (25) 66.7 (2)	80.6 (104)^f^ 33.3 (1)	10.5 (126) 42.4 (14)	89.5 (1,073)^f*^ 57.6 (19)
Combined *n =* 7,281	< 2019 2019+	*n =* 597		*n =* 571		*n =* 6,113	
		16.6 (94) 37.9 (11)	83.5 (474)^c*^ 62.1 (18)	10.3 (54) 19.2 (9)	89.7 (470)^c^ 80.9 (38)	6.8 (391) 26.6 (94)	93.2 (5,368)^c*^ 73.5 (260)

^*^p-value < 0.05, ^c^Chi-square test, ^f^Fisher's exact test.

### 3.3. Impact of the COVID-19 pandemic on adherence to ART

Table 3 shows the estimates of the odds of viral detectability (a measure of adherence to ART) among the adult PLWHIV who were initiated on ART after the U=U campaign initiative was launched in Zambia in 2019, a period largely characterized by the COVID-19 pandemic, compared to those who started before the campaign initiative was launched stratified by the type of first-line ART regimen and ART dispensing interval. Stratified multivariable logistic regression modeling was used to generate the odds of detectable viral load (≥200 copies/ml).

**Table 3:** Stratified and combined adjusted analysis to determine the impact of the COVID-19 pandemic on viral detectability (a measure of adherence to ART) using multivariable logistic regression.

**Type of regimen**	**ART Start Year**	**Dispensing interval (Months)**						**Overall**
		**One**		**Three**		**Six**		
		**OR (95% CI)**	**P-value**	**OR (95% CI)**	**P-value**	**OR (95% CI)**	**P-value**	**OR (95% CI)**
TLD/TafED^*^	< 2019 2019+	Ref 2.51 (1.31, 9.03)	0.012	1 2.38 (0.95, 5.98)	0.064	1 4.75 (3.52, 6.41)	< 0.001	1 4.26 (3.25, 5.58) ^**^
TLE/Atripla^*^	< 2019 2019+	Ref 1.74 (0.27, 11.30)	0.564	1 7.54(0.49,115.8)	0.147	1 4.67 (2.16, 10.08)	< 0.001	1 4.13 (2.06, 8.29) ^**^
Combined^**^	< 2019 2019+	Ref 2.97 (1.25, 7.05)	0.014	1 2.63 (1.13, 6.12)	0.025	1 4.62 (3.51, 6.10)	< 0.001	1 4.14 (3.22, 5.31)^***^

^*^Having adjusted for sex, age, CD4, and WHO clinical stage. ^**^Adjusted for sex, age, CD4, WHO clinical stage, and the type of first-line ART regimen. ^***^Overall estimates between U=U campaign categories while controlling for sex, age, CD4 count, WHO clinical stage, type of first-line regimen, and ART dispensing interval. Refer to Figure 2 for the margins plot of these overall adjusted estimates. TLD (TDF/3TC/DTG): Tenofovir disoproxil fumarate/Lamivudine/Dolutegravir, TafED (TAF/FTC/DTG): Tenofovir alafenamide/Emtricitabine/Dolutegravir, TLE (TDF/3TC/EFV): Tenofovir disoproxil fumarate/Lamivudine/Efavirenz, and Atripla (TDF/FTC/EFV): Tenofovir disoproxil fumarate/Lamivudine/Efavirenz.

After controlling for the effect of other covariates (age, sex, CD4 cell count at ART initiation, and WHO clinical stage at the start of ART), the odds of detectable viral load remained significantly higher among adult PLWHIV who were initiated on ART after the U=U campaign initiative was launched in Zambia and were on a monthly (AOR = 2.51, 95% CI 1.31–9.03, *p*-value = 0.012), or 6-monthly (AOR = 4.75, 95% CI 3.52–6.41, *p*-value < 0.001) dispensing of a dolutegravir-based first-line regimen and those who were on a 6-monthly dispensing of an efavirenz-based first-line regimen (AOR = 4.67, 95% CI 2.16–10.08, *p*-value < 0.001) compared to their counterparts who were initiated on ART before the campaign was launched in Zambia. Overall estimates also showed similar results (AOR = 4.14, 95% CI 3.22–5.31). Furthermore, when we considered both types of first-line regimens, the odds of detectable viral load remained significantly higher among post-U=U campaign ART initiators compared to their counterparts who started earlier and were on a monthly dispensing interval (AOR = 2.97, 95% CI 1.25–7.05, *p*-value = 0.014), 3-monthly dispensing interval (AOR = 2.63, 95% CI 1.13–6.12, *p*-value = 0.025) or a 6-monthly dispensing interval (AOR = 4.62, 95% CI 3.51–6.10, *p*-value < 0.001). Even though this positive association was maintained when we considered adult PLWHIV who were taking an efavirenz-based first-line regimen every month or every 3 months, and those who were taking a dolutegravir-based first-line regimen every 3 months, the findings were not statistically significant.

### 3.4. Viral load detectability among adult PLWHIV on ART during the COVID-19 pandemic

**Figure 2:**
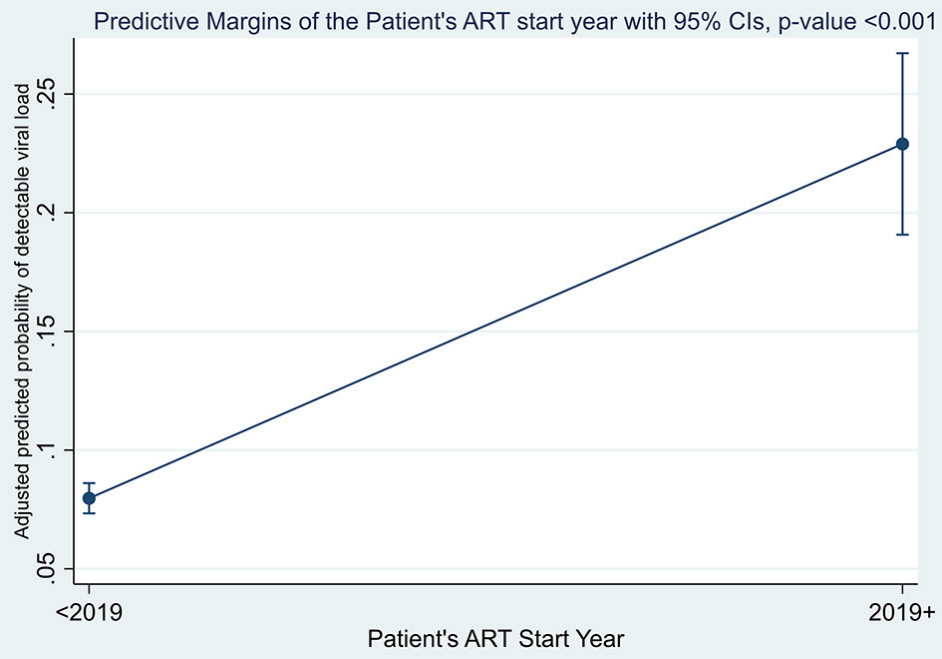
Margins plot on overall viral detectability among adult PLWHIV by campaign categories.

The margins plot for the overall adjusted estimates of detectable viral load between the two campaign categories was explored and the findings show that adult PLWHIV who were initiated on ART after the launch of the U=U campaign initiative in Zambia were more likely to have a detectable viral load than their counterparts who were initiated on ART before 2019, as shown in Figure 2. The margin probabilities were significantly different from zero (*p*-value < 0.001).

## 4. Discussion

We found a relatively high proportion of people with detectable viral load (≥200 copies/ml) in the study population and this was most concentrated among adult PLWHIV who started ART during the COVID-19 pandemic amid the U=U campaign initiative compared to their counterparts who initiated ART before the campaign was launched in Zambia in 2019. This proportion was consistently similar in all the population demographic strata. This is similar to findings published from other regions, such as Guatemala in Central America (18) and the Dominican Republic in the Caribbean region (19). This suggests that such outcomes are attainable in diverse settings. Furthermore, a high level of detectable viral load was found among adult PLWHIV engaged in HIV care at ten health centers in Rwanda (20). To maintain an undetectable viral load (≤ 200 RNA copies/ml), PLWHIV are supposed to diligently take ART medications daily as prescribed. Otherwise, viral detectability and HIV transmission remain high risks (7). Disruptions in clinic attendance and personal ART supplies that were characteristic of the COVID-19-induced lockdown significantly impacted patients' adherence to ART drugs (21). Similarly, we observed disparities in the levels of viral detectability between adult PLWHIV who were initiated on ART during the COVID-19 pandemic period (2019+) and those who were initiated on ART before that period, suggesting that the pandemic had a negative impact on patient's adherence to daily prescribed ART medications.

There may be some selection biases that may be inherent in our study. This may arise from the fact that this is already a selected group attending a specific health facility. However, we have no reason to assume that any form of selection could be important in explaining our observations. This is because firstly, the data used in this study were HIV program data for all the adult PLWHIV receiving ART from the AIDC, UTH in Lusaka, Zambia which is the main referral ART center in the country. Therefore, this population could serve as a de facto target population of all PLWHV thus suggesting that the study power is optimal. In addition, the possibility of any other confounding associated with selection was further minimized in that all analyses were controlled for interaction. In particular, the logistic regression technique was used to model the effect of the COVID-19 pandemic on the odds of the patient's viral detectability, a measure of ART adherence in this study. Both stratified and combined adjusted analyses were done to control for confounding and effect modification biases in our measurements. Therefore, we have no ground to attribute these findings to bias.

In general, we are cognizant that attributability is difficult to ascertain. However, we are also aware that the major event that happened on a large scale during the period data was collected, soon after the U=U campaign initiative was launched in Zambia, was the COVID-19 pandemic. The effects of the COVID-19 pandemic on HIV care are well documented (22–24) and we think more will continue to be documented. In our study, therefore, the increased likelihood of having a detectable viral load among adult PLWHIV who were initiated on ART during the pandemic period was most likely due to HIV program services-related disruptions brought about by the COVID-19 pandemic. Implementation of quarantine, social distancing, and community containment measures during the COVID-19 pandemic may have reduced access to routine HIV services, such as access to ART refills, hindering adult PLWHIV's ability to adhere to ART medications. This ultimately may have led to the increased risk of having a detectable viral load among those who started ART during the pandemic period. This is because the initial planning of HIV programs did not take into account the COVID-19 pandemic.

Treatment duration has been found to have a mixed effect on viral suppression. Studies conducted in Uganda and Kenya found no association between the two (25, 26). Even though studies conducted in Ghana and Zimbabwe found that longer duration was associated with higher levels of viral suppression, the cutoff duration points were 3 years and 4.5 months, respectively (27, 28). From this, we can conclude that the cutoff duration points are inconsistent and there are no proper scientific explanations for the comparable durations. In the context of our study, a patient on therapy for at least 6 months was expected to achieve an undetectable viral load if they took their medicine as prescribed (17). It is for this reason that we only included patients who had been on treatment for at least 6 months. We, therefore, attributed the difference in viral load detectability (used as a proxy for poor adherence to ART) to COVID-19-related disruptions as the major event that happened on a large scale during the study's period.

Evidence from sub-Saharan Africa has shown that multi-month dispensing of ART medications improves adherence to ART and viral suppression compared to the standard monthly refill interval (29–31). However, this is somewhat different from what was observed where in all the strata people who started ART during the pandemic period were consistently at a higher risk of having a detectable viral load than those who started before the pandemic, irrespective of ART refill intervals. There could be many explanations for this but we are persuaded to think that the COVID-19 pandemic is one of them. This is because widespread health system disruptions due to COVID-19 in various settings were reported (32), resulting in reduced health service utilization in general and for various reasons, ranging from negative myths on how the infection spreads to a general fear of contracting the infection, including fear of being tested and fear of being vaccinated (33–36). The emergence of the COVID-19 pandemic masked the effects of the U=U campaign because of the kind of COVID-19 control measures being implemented, which included quarantine, controlled local travel or mobility, social distancing, and limited access to health facilities for ART refills due to fear of exposure to the virus. Furthermore, in order to optimize the healthcare workforce and services during the COVID-19 pandemic, limited resources from previously existing health programs, such as the U=U campaign initiative, were diverted to the fight against the COVID-19 pandemic (37, 38).

However, we think that the effect of COVID-19 is not infinite given the stability in the spread that is already being experienced globally. Therefore, our observations should not pause the continued effect of the U=U campaign. Instead, we believe the campaign should be further strengthened and revived because it has a place in the HIV response program, especially as the world is strategizing for functional endgame approaches in the fight against HIV. This is because the U=U campaign initiative, which was first launched by the Prevention Access Campaign in 2016, bears the message that PLWHIV on ART for at least 6 months and who take ART medications daily as prescribed achieve and maintain an undetectable viral load (< 200 copies/ml), and thus have effectively no risk of sexually transmitting the virus through unprotected vaginal or anal sex to their HIV-uninfected sexual partners (5). Further evidence suggests that when healthcare providers discuss this U=U message with PLWHIV, there is further improved ART adherence and viral suppression (8).

In Zambia, the COVID-19 pandemic started soon after the launch of the U=U campaign initiative (10). This could explain why, despite the campaign's launch in mid-2019, its effect on patients' adherence to medication seems to have been masked by the pandemic. This generalized effect on the health system invariably affected the ART program too, possibly diluting the effect of the U=U campaign that had been intended to improve adherence. Additionally, the finding that adherence was affected irrespective of ART regimen strata, including for those on the first line, was not surprising because the impact of COVID-19 was generalized irrespective of population subgroups (21). This uniformity in the interruption of HIV care services brought about by the COVID-19 pandemic might be the most important factor in explaining the observations we reported in this paper.

### 4.1. Study strengths and limitations

This study has several strengths. It employed a large sample size (*n* = 7,281). This powered our study to detect the small differences in effect sizes between the groups. Furthermore, this study used the whole data set of the adult PLWHIV on ART at the University Teaching Hospital. Due to this sampling method, selection bias was not large enough to impact the study results. Both stratified and combined adjusted analyses were done to control for confounding biases in our measurements. Therefore, we have no ground to attribute these findings to bias.

However, the following should be considered while interpreting the abovementioned findings. First, generalizability is difficult to ascertain as this was a single-center study and only included patients with available data on variables of interest. This could have affected the true estimates of the effect sizes. Secondly, this was a cross-sectional study, therefore, we could not establish causality. Furthermore, we did not have data on behavioral and other patient-related factors, such as comorbidities and mental health. These factors might explain some of the observed differences in viral suppression among many subgroups in our cohort.

Nonetheless, our findings illustrate how exposed program responses, such as the U=U campaign initiative, are to external shocks, especially in already weakened health systems such as the one in Zambia. This calls for the need to create resilient programs to withstand the effect of external disruptions.

## 5. Conclusion

We report a high proportion of people with detectable viral load in the study population irrespective of ART refill interval and type of ART regimen. However, this was most concentrated among adult PLWHIV who started ART amidst the U=U campaign initiative when compared to those who initiated ART before the campaign was launched in Zambia in 2019. This observed disparity suggests the inherent impact of the COVID-19 pandemic on the adherence to ART among adult PLWHIV in Lusaka, Zambia. This further illustrates how exposed program responses are to external shocks, especially in already weakened health systems. Therefore, our observations provide warning signs and a call for the urgent creation of program response buffers to external shocks as well as a need to reflect on creating resilient program-specific strategies to minimize the effect of external disruptions. One such possibility is the implementation of a workforce strategy, such as the system not using redistribution of the health workforce for firefighting as it exposes the system further. Without redirecting the workforce, perhaps the already existing U=U campaign initiative, even amid the pandemic, could have been sustained. As countries race to enter the path to the elimination of HIV, specifically eMTCT and ending HIV/AIDS as a public health threat by 2030, there is a need to create resilient health systems with specific program-to-program innovations that are context-specific and driven by local evidence.

## Data availability statement

The original contributions presented in the study are included in the article/Supplementary material, further inquiries can be directed to the corresponding author.

## Ethics statement

The studies involving human participants were reviewed and approved by the University of Zambia Biomedical Research Ethics Committee (UNZABREC) (Ref. no. 2099-2021). Written informed consent for participation was not required for this study in accordance with the national legislation and the institutional requirements.

## Author contributions

PK: conceptualization, data curation, formal analysis, investigation, methodology, and writing—original draft. FMN: conceptualization, data curation, refinement, and writing—review and editing. CM: overall oversight, conceptualization, formal analysis, methodology, supervision, and writing—review and editing. All authors contributed to the article and approved the submitted version.
